# The Impact of ApoE e4 Allele in the Effects of Physical Activity Interventions on Major Mobility Disability Incidence

**DOI:** 10.1093/geroni/igaa057.475

**Published:** 2020-12-16

**Authors:** Erta Cenko, Todd Manini

**Affiliations:** 1 University of Florida, Gainesville, Florida, United States; 2 The University of Florida, Gainesville, Florida, United States

## Abstract

The apolipoprotein E (ApoE) e4 polymorphism is traditionally linked to increased rates of cognitive impairments and Alzheimer’s Disease. Recently, however, studies have connected ApoE e4 to self-reported mobility disability in those without cognitive impairment. We aimed to estimate the extent to which ApoE status is associated with incident major mobility disability (MMD) and whether it modifies the known beneficial effects of physical activity on incident MMD. Data were from 1,372 participants 70+ years old at risk for mobility impairments, enrolled in The LIFE Study. Participants were randomized to a structured physical activity (PA) or a health education program. The primary outcome was MMD, defined as the inability to walk 400 meters and measured every 6 months for an average follow-up period of 2.6 years. We used proportional hazards regression to examine the main effect of ApoE allele status and interaction with intervention group on MMD incidence. Cumulative MMD incident rates were similar in ApoE-e2 (31.46%), ApoE e3 (33.41%), and ApoE-e4 (33.33%) carriers. Compared to the common ApoE-e3, MMD risk was similar in ApoE-e2 (hazard ratio [HR], 0.945 [95%CI, 0.70-1.27], P=0.71) and ApoE-e4 carriers ([HR], 1.187 [95%CI, 0.95-1.48], P=0.41). Additionally, ApoE carrier status did not modify the positive effect that a PA intervention had on MMD risk (interaction p-value > 0.20). These results suggest that ApoE carrier status is not associated with incident mobility disability. ApoE carrier status does not impact the effect a structured PA program has on reducing the risk of MMD in older adults.

